# Teaching in Geriatrics: Is structured written feedback effective for lectures

**DOI:** 10.1093/geroni/igab046.2803

**Published:** 2021-12-17

**Authors:** Theresa Pohlmann, Klaus Hager, Volker Paulmann, Sandra Steffens

**Affiliations:** 1 Medical School Hannover, Hannover, Niedersachsen, Germany; 2 Medical School of Hanover, Hannover, Niedersachsen, Germany

## Abstract

Background: Although there have been discussions about traditional lecturing, lectures are still largely a widespread concept of knowledge transfer. Therefore, it is important to constantly review and evaluate this format. The aims of this study were to analyze which effect a criteria-based written feedback has on the lecture course in geriatrics as an alternative to the conventional student evaluation. Furthermore, we wanted to investigate what kind of impact structured feedback has on lecturers in terms of content, organization and quality. Methods: The study was a prospective longitudinal analysis. The 34 lectures on the subject of geriatrics were analyzed over two cohorts using a standardized evaluation sheet. The assessment was carried out on a 5-point-scale using a 22-item feedback instrument. After the first evaluation, each lecturer received an individual evaluation with strengths and suggestions for improvement. In the second cohort the lecture series was evaluated again, and individual feedback was sent. Results: In six of 22 sub-categories the improvement was significant. The most significant improvement was made in terms of content/structure with an increase from 3.4 to 4.3 points. Conclusion: This study shows that significant improvement in teaching is possible by means of individualized written feedback for the lecturers and that students perceive the resulting improvements positively. Our results suggest that the implementation of these feedback instruments in other modules might improve their teaching as well.

